# The need for new metrics in the Anthropocene era

**DOI:** 10.3389/fpubh.2022.935743

**Published:** 2022-08-04

**Authors:** Paolo Vineis, Lorenzo Mangone

**Affiliations:** ^1^School of Public Health, Imperial College, Medical Research Council (MRC) Centre for Environment and Health, London, United Kingdom; ^2^Sant'Anna School of Advanced Studies, Pisa, Italy; ^3^Regenerative Society Foundation, Parma, Italy

**Keywords:** climate change, regenerative economy, sdg, EROI, co-benefits

## Abstract

A limitation in the discussion concerning climate change is the large degree of separation between scientific, economic, and technological approaches to tackle the crisis. This issue is most noticeable when considering the lack of metrics to measure the impact of different productive sectors on both the environment and the health of the population. The best-known attempt to measure these repercussions has been the introduction of the Environmental, Social and Governance (ESG) ratings for bonds. However, this rating system suffers from a lack of transparency and standardization. Moreover, it does not offer insights on the health impact and the regenerative effort of the evaluated bonds. Thus, we think it is necessary to introduce new metrics, focusing on at least four dimensions: circularity, climate change, biodiversity and health (including well-being). A sector that needs a special consideration is that of energy. To better compare different energy sources, we propose to adjust metrics such as the Energy Return on Investment (EROI) or the energy intensity metrics to include the negative health effects and the environmental degradation associated with producing energy. A similar index of return on investment corrected for health impacts may be considered to evaluate food production as well. Hyper-analytical and extremely focused approaches have dominated the discussion around the environmental crisis. We believe that a more inclusive approach is now needed, to highlight the potential co-benefits of different strategies, especially those that promote regeneration and a truly circular economy.

## Introduction

The years to come will present pressing and dramatic challenges in public health, due to the impact on human health of climate change, loss of biodiversity, and environmental degradation. The scientific and technological responses have been so far characterized by a lack of coordination and integration, essentially working in silos. For example, only recently preparedness to pandemics has been related to the “One Health” concept and to environmental changes such as deforestation or intensive animal breeding. Approaches and technologies for public health to tackle such challenges are outdated: the connections across the *natural, biological, social, economic, and cultural capitals* are not systematically considered. One particularly important gap is represented by the lack of economic metrics to measure the impact of different productive sectors and activities on health and the environment. Though the concept of “co-benefits” is now appreciated - i.e., joint efforts across sectors (such as different Ministries) to address health and climate change mitigation together (1, 2) -, concepts such as circular economy and environmental impact are not yet fully and properly embedded in economics and finance.

Some steps forward have been made recently with the introduction of ESG ratings. Bonds are now labeled according to the ESG (Environmental, Social, and Governance) scheme impacts. The ESG rating typically is a score summarizing intangible assets within the enterprise, essentially functioning as a form of corporate *social credit score*. Such intangible assets are now considered to comprise an increasing percentage of future enterprise value. They can be used in multiple instances, ultimately measuring features representing sustainability and societal impact of a company or business. Between 2015 and 2019 there has been a 525% increase in the assets of companies that refer to ESG ratings, a trend that shows how these measures are to be taken extremely seriously. However, ESG ratings are emitted in the form of certificates by private agencies, and as far as we know there is little public control over their (non-economic) value. It seems that the data summaries provided for the analyses that generate the ratings are produced by the rated companies themselves, though they seem to be at least partially verifiable. The current lack of transparency, i.e., of a rigorous control on the accuracy of the data, opens the way to so-called greenwashing. Thus, it is of paramount importance to introduce a set of metrics and standards in the field of ESG, such as those traditionally provided for similar purposes by ISO (International Organization for Standardization). In addition to transparency and standardization, few limitations make this rating system suboptimal: first, ESG refer essentially to sustainability rather than regeneration, and second, they are limited in not including relevant themes such as health and well-being.

Why do we mention regeneration beyond sustainability? The current “extractive” economy cannot realistically restore the damage it has inflicted to the planet. If we consider the Earth Overshoot Day, i.e. the day in which the resources available for the year have been exhausted, it is clear that we need to go much beyond mitigation. In 2021 this day fell at the end of July, signaling a severe depletion of the resources our planet is able to produce in a single year; therefore, we need to start regenerate the resources that decades of exploitative practices have progressively destroyed. Consequently, indicators that have the ambition of promoting truly sustainable practices, such as the ESG, should not simply offer mild reassurances about a decrease in resource extraction. To be able to properly evaluate the social and environmental impact of an enterprise some completely novel metrics need to be proposed. An example regarding the climate impact of an agricultural enterprise is the measurement of CO2 absorption via agricultural carbon sinks, a regenerative practice.

The second shortcoming that we found with ESG is the lack of consideration of the health impact. Any factory that aims at ESG certification could implement a CO2 abatement strategy that does not take into account the parallel emissions of air pollutants. Carbon capture and storage (CCS) strategies are becoming available as a transitional measure toward a zero-emission future, however they seem to ignore that the higher fuel consumption they require would determine an increase in particulate matter (PM) emissions (3). Factories that simply adopt CCS without considering air pollution would therefore be judged virtuous (by contributing to climate change mitigation) while simultaneously having an increased impact on population health, as PM is responsible for conditions like asthma, cardiovascular diseases and cancer. However, it has been suggested that prioritizing intersectional actions that take into account both disease prevention and climate change mitigation can have a positive impact not only on society but also on the economy (4).

## Discussion

### Characteristics of the new metrics

We believe that new metrics that can promote regeneration should include at least four dimensions: *circularity, climate change (carbon stock), biodiversity, and health including well-being*. Obviously, this classification is purely tentative and could be different, e.g. by including climate change and biodiversity under the natural capital concept, but fine-tuning is not the purpose of our paper. Multiple dimensions are needed. For example, a company can have a virtuous overall balance, in which the different segments - from raw material acquisition, packaging, storage, transportation, down to retail of end products - assure circularity and restoration of the natural capital, but produce unhealthy goods that impact on human health. Well-being is still another issue, even less explored than health impacts. Companies can impact the health on many different scales, for example they can facilitate a sustainable lifestyle through action limited in scope such as the promotion of plant-based food at the cafeteria, or the facilitation of active transportation for personnel. Actions such as a better management of waste and improvements in heat retention are larger in scope, affecting emissions and health impact at the whole factory level and beyond. Finally, companies can contribute to the well-being of the population at large: for example, local distribution based on short value chains allow consumers to purchase goods more easily, lead to a reduction in the use of cars (as opposed to reaching distant hypermarkets) and may improve subjective well-being and mental health. A real-life example is the juxtaposition between the old model of a town, in which people were used to walk and food was purchased in the surrounding countryside, and the new model that requires the use of cars and food is imported from a long distance.

In Table 1, we have reported a first and tentative list of potential indicators and their measurements while Table 2 offers a breakdown of the metrics concerning circularity present in Table 1. Circularity has been defined as “a model that decouples economic activity from linear material flows, thus going beyond “doing less bad” to regenerating planet and society. Its main objectives are to eliminate waste and pollution and promote the circulation of products and materials at their highest value”^1^.

**Table 1:** Indicators of environmental impact of entities and their measurements.

**Indicator**	**Measurements**
**Climate resilience**	
Negative net CO2eq	Emissions Measures of CO2 or other GHG emissions/absorption
**Natural capital restoration**	
Promotion of biodiversity value	Restoration of high conservation value
Promotion of soil fertility	Chemical and biological parameters of soils
Restoration of high conservation value (HCV) areas	Enlargement of highly conserved areas
Restoration of clean water	Restoration of clean water amount and availability of water for humans and other species (safety, physico-chemical-biological properties)
**Circularity**	
Zero waste production / Closed loop	Circularity to be included in industrial design
Safety / Toxic substances elimination (Stockholm POP convention)	Indicators of progress according to goals set by EEA, EPA, etc..
**Well-being: health and happiness**	
Public health restoration	Good examples can be found among Lancet countdown indicators
Fair labour promotion Communities and local populations Positive impact	Indicators of satisfaction, quality of life, participation in societal life and policy-making, mental health

**Table 2:** Breakdown of measurements of circularity.

Longevity: Designed for maintenance, longevity and durability in such a way that encourages longer use than the industry standard in practice (e.g., promote repair rather than replacement, timeless design, durable material choices)
Reusability: Designed for multiple uses in such a way that ensures actual reuse in practice (e.g., secondary markets, packaging reuse systems, standardized design)
Repairability: Designed for repair in such a way that uses existing systems for repair in practice (e.g., network of repair shops, your own repair service). Examples of design choices are: modular design / built in predictive maintenance sensors, repair diagnostics etc. / designed with right to repair by third parties / designed for remanufacturing / using standardized components across a sector
Recyclability: Designed in such a way that uses existing recycling systems that operate in practice and at scale
Simplicity and Disassembly: Designed in a way that it is separable in recyclable parts (e.g., low materials complexity, modular design, reversible connections, ease of separating materials)

### Complex indicators

Properly evaluating the environmental impact of entities requires more than the sole ESG ratings, since these are relevant mainly to the financial world and the assessment of companies. Therefore, metrics need to be developed for different purposes. Specifically, the concept of energy needs a special mention as tackling it is instrumental for the current environmental and public health crisis.

Ultimately most of the energy sources on earth, both those involved in keeping our industrialized society working and those that we require to keep on living as humans, derive from the sun. The main difference between a source like the natural gas used in a thermoelectric power plant and the grains we consume in our diet is timing and efficiency. Fossil fuels are the product of millions of years of transformation of vegetable residues that originally came from photosynthesis, i.e., sun energy. Similarly, every nutrient we assume with food is related, directly or indirectly, to photosynthesis, be it the intake of vegetables or fruits, or of meat that is based on animal grazing.

A useful metric to evaluate energy is Energy Return on Investment (EROI)^2^, which is the difference between the energy available for use and the energy invested to produce energy, for each source. Oil had a very high EROI in the past, that now is decreasing due to depletion of the most accessible sources. This fact is leading to an increase in the energy investment required to find and refine new deposits. Other sources, such as biomass in temperate areas, have very low EROI. The same concepts apply to human nutrition: in Western societies each of us contributes very little (and in indirect ways) to the production of energy incorporated into the food we eat (hence the problem of obesity), while in LIC the EROI for food may be negative (more energy is used than is available, hence malnutrition).

We can propose to develop a new metric that is “EROI-augmented,” including climate impact and health impact. Incorporation of health means for example considering the effects of pollution from fossil fuels, that is an externality so far not considered in EROI. Similarly, the parallel concept of energy intensity, a measure of the amount of energy produced for a fixed amount of money, should take into account the cost of treatment of diseases that are partially attributable to pollution and climate change. It would be disingenuous not to consider that relying on fossil fuels (commonly considered a cheap source with a high EROI) causes stress on the health system that leads to additional energy intakes and significant costs.

Thus, a new metric to evaluate energy other than EROI could be:


$$\begin{array}{l} EROI_{aug}=\frac{Energy\;available\;to\;society}{Energy\;invested\;in\;production+negative\;health\;effects+environmental\;degradation}. \end{array}$$

In agriculture this would translate into food production, that is another kind of transformation of energy in itself, with a metric like:


$$\frac{{\text{Energy}}^{\text{*}}\text{ made available to the population + positive health effects}}{\text{energy needed to produce it + negative health effects + environmental impact including loss of biodiversity}}$$

^*^Calories plus nutrients and micronutrients.

We believe that these types of metrics could be particularly important for the policy makers and should be considered especially by governmental and supranational institutions (e.g., the European Union). Having a quick and effective way to compare energy sources that simultaneously provides insights about future consequences of choosing one source over another would allow for long-sighted and evidence-driven decisions. Likewise, the metric representing food production could help in justify policies that could strongly impact the health of the populations while simultaneously tackling climate change and its consequences.

An important attempt to include climate change mitigation into nutritional guidelines, in addition to the impact on health, are the EAT-Lancet guidelines, also called “diet for the Anthropocene” (5). The guidelines were successfully used to assess the potential co-benefits from shifting to more sustainable diets, showing that, in a European population, a better adherence to the EAT-Lancet diet could prevent a large number of deaths in a 20-year risk period (6). Criticisms have been raised to these guidelines, from the Western-centric bias inherent in the guidelines to the virtual absence of consideration of the specific needs of large populations, from sick people to children and elders; therefore, much work still needs to be done.

For more detailed indicators of the relationships between climate change and health, an excellent set is represented by the Lancet Countdown indicators^3^, that have been proposed to monitor how the Paris agreement goals are met in the course of time and are showing that so far, we have accomplished very little of what was needed.

## Conclusion

We are at high time with tackling the environmental crisis. Several mistakes have been made so far, including an approach that was hyper-analytical and very narrow in scope, for example chemicals are evaluated one at a time for their toxicity (typically, carcinogenicity for “legacy” chemicals) without any consideration for synergic effects. We now realize that the impacts go much beyond human health and a more comprehensive approach is needed. Another mistake has been to overlook the need for circularity. Not only because the extractive economy leads to a multiplicity of negative impacts, including residues and wastes, but also because circularity has been interpreted in a limited way as circular management of industrial waste, i.e., just at the end of the productive system. Instead, it should be embedded into industrial design itself (starting from raw materials) and include all steps.

What we propose here is only a general framework to allow the assessment of environmental and health impacts of human activities, to reverse the “earth overshoot” we have caused. The framework needs to be populated with good indicators that can be used practically to improve regenerative performances by avoiding greenwashing.

## Data availability statement

The original contributions presented in the study are included in the article/supplementary material, further inquiries can be directed to the corresponding author/s.

## Author contributions

PV contributed to conception of most of the theories and wrote the first draft of the manuscript. LM expanded on the original ideas and wrote new sections of the manuscript. All authors contributed to manuscript revision, read, and approved the submitted version.

## Conflict of interest

Author LM was employed by the Regenerative Society Foundation. The remaining author declares that the research was conducted in the absence of any commercial or financial relationships that could be construed as a potential conflict of interest.

## Publisher's note

All claims expressed in this article are solely those of the authors and do not necessarily represent those of their affiliated organizations, or those of the publisher, the editors and the reviewers. Any product that may be evaluated in this article, or claim that may be made by its manufacturer, is not guaranteed or endorsed by the publisher.

## Footnotes

^1^Available online at: https://ellenmacarthurfoundation.org/articles/design-and-the-circular-economy.

^2^Available online at: https://nyaspubs.onlinelibrary.wiley.com/doi/epdf/10.1111/j.1749-6632.2009.05282.x.

^3^Available online at: https://www.thelancet.com/journals/lancet/article/PIIS0140-6736(21)01787-6/fulltext.
